# Mucopolysaccharidosis VI in cats – clarification regarding genetic testing

**DOI:** 10.1186/s12917-016-0764-y

**Published:** 2016-07-02

**Authors:** Leslie A. Lyons, Robert A. Grahn, Francesca Genova, Michela Beccaglia, John J. Hopwood, Maria Longeri

**Affiliations:** Department of Veterinary Medicine and Surgery, College of Veterinary Medicine, University of Missouri – Columbia, Columbia, MO 65211 USA; Veterinary Genetics Laboratory, School of Veterinary Medicine, University of California - Davis, Davis, CA USA; Department of Veterinary Medicine, University of Milan, Milan, Italy; Ambulatorio Veterinario, Lissone, Monza e Brianza Italy; Lysosomal Diseases Research Unit, South Australian Health and Medical Research Institute, Adelaide, Australia

**Keywords:** *ARSB*, DNA, Feline, *Felis silvestris catus*, Genetic testing, *N-acetylgalactosamine-4-sulfatase*, MPS VI

## Abstract

The release of new DNA-based diagnostic tools has increased tremendously in companion animals. Over 70 different DNA variants are now known for the cat, including DNA variants in disease-associated genes and genes causing aesthetically interesting traits. The impact genetic tests have on animal breeding and health management is significant because of the ability to control the breeding of domestic cats, especially breed cats. If used properly, genetic testing can prevent the production of diseased animals, causing the reduction of the frequency of the causal variant in the population, and, potentially, the eventual eradication of the disease. However, testing of some identified DNA variants may be unwarranted and cause undo strife within the cat breeding community and unnecessary reduction of gene pools and availability of breeding animals. Testing for mucopolysaccharidosis Type VI (MPS VI) in cats, specifically the genetic testing of the L476P (c.1427T>C) and the D520N (c.1558G>A) variants in *arylsulfatase B (ARSB)*, has come under scrutiny. No health problems are associated with the D520N (c.1558G>A) variant, however, breeders that obtain positive results for this variant are speculating as to possible correlation with health concerns. Birman cats already have a markedly reduced gene pool and have a high frequency of the MPS VI D520N variant. Further reduction of the gene pool by eliminating cats that are heterozygous or homozygous for only the MPS VI D520N variant could lead to more inbreeding depression effects on the breed population. Herein is debated the genetic testing of the MPS VI D520N variant in cats. Surveys from different laboratories suggest the L476P (c.1427T>C) disease-associated variant should be monitored in the cat breed populations, particularly breeds with Siamese derivations and outcrosses. However, the D520N has no evidence of association with disease in cats and testing is not recommended in the absence of L476P genotyping. Selection against the D520N is not warranted in cat populations. More rigorous guidelines may be required to support the genetic testing of DNA variants in all animal species.

## Background

Genetic testing represents one of the most important hot topics in the field of veterinary small animal practice [1, 2]. The discovery and release of DNA-based diagnostics has tremendously increased, and the impact of these tests on animal breeding and health and population management is significant. All DNA tests need extensive “vetting” by the research community to document sensitivity, specificity and correlation with disease since the downstream consequences of publishing ambiguous or benign genetic variants have the potential to dramatically impact the genetic health of a population.

One recent example of a controversial DNA test in cats was the A31P and A74T variants in *myosin-binding protein C3* (*MYBPC3)* for hypertrophic cardiomyopathy (HCM) [3]. The A31P variant has a strong correlation with disease – as an indicator of risk, with a late-onset Mendelian autosomal dominant transmission, in Maine Coon cats. However, an A74T variant was presented in an abstract and suggested as causative for the same disease, which elicited a strong debate in the veterinary community [4]. Genetic testing was initiated by several commercial laboratories in worldwide cat breed populations. However, data strongly indicated that the A74T was a common polymorphism in a large number of breeds, and not significantly correlated to HCM [5]. Therefore, the A74T DNA variant testing was subsequently removed from many commercial laboratory offerings and no breeding decisions should be influenced by the presence or absence of this variant in a domestic cat.

A similar situation has recently developed for the genetic testing of mucopolysaccharidosis Type VI (MPS VI), an *arylsulfatase B (ARSB*) variant in cat breeds [6–9]. The published phenotypes and implications of the L476P (c.1427 T > C) variant in *ARSB* that causes a severe form of MPS VI have been recognized for many years [10]. However, a second variant of *ARSB* was identified within the same experimental feline colony [11, 12]. Several laboratories now offer this MPS VI D520N “mild” DNA test to the cat breeding community. Unlike HCM, veterinarians and cat owners are less familiar with MPS VI, both in regards to clinical presentation and disease course.

Unarmed with the proper knowledge and poor availability of genetic counselling, breeders have become alarmed since many cats have been genotyped as carriers or homozygous for the D520N *ARSB* variant. A wave of genotyping in Birmans for the D520N variant has escalated the concern with breeders, who are now attributing pyometra, pregnancy loss, infertility and other health problems as a result of the variant (Personal communication – several authors). Hence, the impetus for this positional paper and a perfect example of the questions the scientific, veterinarian and breeder community will frequently address in the future. The questions are: does this mutation cause a “real disease”, a “dysfunction” and is it worthy of negative selection and elimination from breeding programs? Does this variant predict a strong association with disease – as a risk indicator? A quick and massive selection for any genetic traits can lead to a drastic reduction of the genetic variability in the breed and the potential emergence of other problems, potentially more devastating, such as the severe form of MPS VI.

The authors consider the *ARSB* D520N variant not causative of a dysfunction or health concern unless present in combination with the *ARSB* L476P variant. Selection against cats with the D520N variant is only warranted if also testing for L476P. However, the L476P variant has not been demonstrated to be present in cat breed and domestic cat populations that have been tested to date. Presented is an evaluation of current genotyping data that supports this position.

### What is mucopolysaccharidosis Type VI (MPS VI)?

The lysosomal storage diseases (LSD) form a diverse group of conditions linked by the common pathological feature of abnormal accumulation of metabolites within cells (for review) [13]. LSD results from a deficiency of an enzyme of one of the lysosomal catabolic pathways. The lysosomes provide a suitable environment for enzyme-catalyzed hydrolysis, keeping other cellular structures protected from the harmful effects of unconfined degradative enzymes. The LSD group is organized into subgroup classifications comprised of more than 30 different diseases based of the metabolic pathways affected and the type of storage identified. Humans with MPS have at least seven different disease entities that include skeletal, cardiovascular, neurological and ocular abnormalities and these same conditions are found in MPS cats [6–8, 14–18].

### MPS VI in humans

In humans, MPS VI, or Maroteaux - Lamy syndrome (OMIM:253200), is a rare autosomal recessive LSD [19]. MPS VI is caused by homozygous or compound heterozygous mutation in *ARSB* (OMIM:611542) [20]. The disease has been estimated to affect 1:200,000–1,500,000 people worldwide [21]. Pathogenic variants of *ARSB* result in reduced or absent activity of the enzyme arylsulfatase B. The reduced activity leads to high urinary excretion and intralysosomal accumulation of large amounts of partially degraded glycosaminoglycans (GAG - previously also known as a “mucopolysaccharide” dermatan sulfate) as well as chondroitin sulfate, N-acetyl-galatosamine and N—acetylgalatosiamine-4,6-disulfate [22, 23]. GAG accumulation results in cell injury with a multi-systemic clinical picture of bone abnormalities, collectively referred to as dysostosis multiplex (disorder of the development of bone, in particular, the ossification), which affects growth, gait, and appearance.

Depending on the age of onset and progression of symptoms, patients with MPS VI have been classified into severe, intermediate, and “attenuated” forms [24]. The severe form of MPS VI is characterized by very early onset and severe progression of symptoms with various skeletal abnormalities (severe dysostosis), including dwarfism, facial dysmorphisms, and joint deformities that affect mobility. Individuals with MPS VI may develop a narrowing of the spinal canal (spinal stenosis) in the neck, which can compress and damage the spinal cord. Death generally occurs during childhood or adolescence.

The attenuated MPS VI form is characterized by very late onset with affected individuals living nearly normal life expectancies. Patients have been described with corneal clouding and joint stiffness [25], or mild dysostosis [26]. The intermediate form shows the middle of the spectrum of phenotypes, however, no fixed criteria for separating these descriptive categories are defined.

Since 1991, nearly 100 genetic mutations had been identified in *ARSB* in patients with MPS VI [20, 27–30] and are cataloged in a database (http://mps6-database.org) [31]. All types of variants can cause all severities of MPS VI and compound heterozygotes can also affect the severity of disease [28].

### MPS VI in cats

**Fig. 1:**
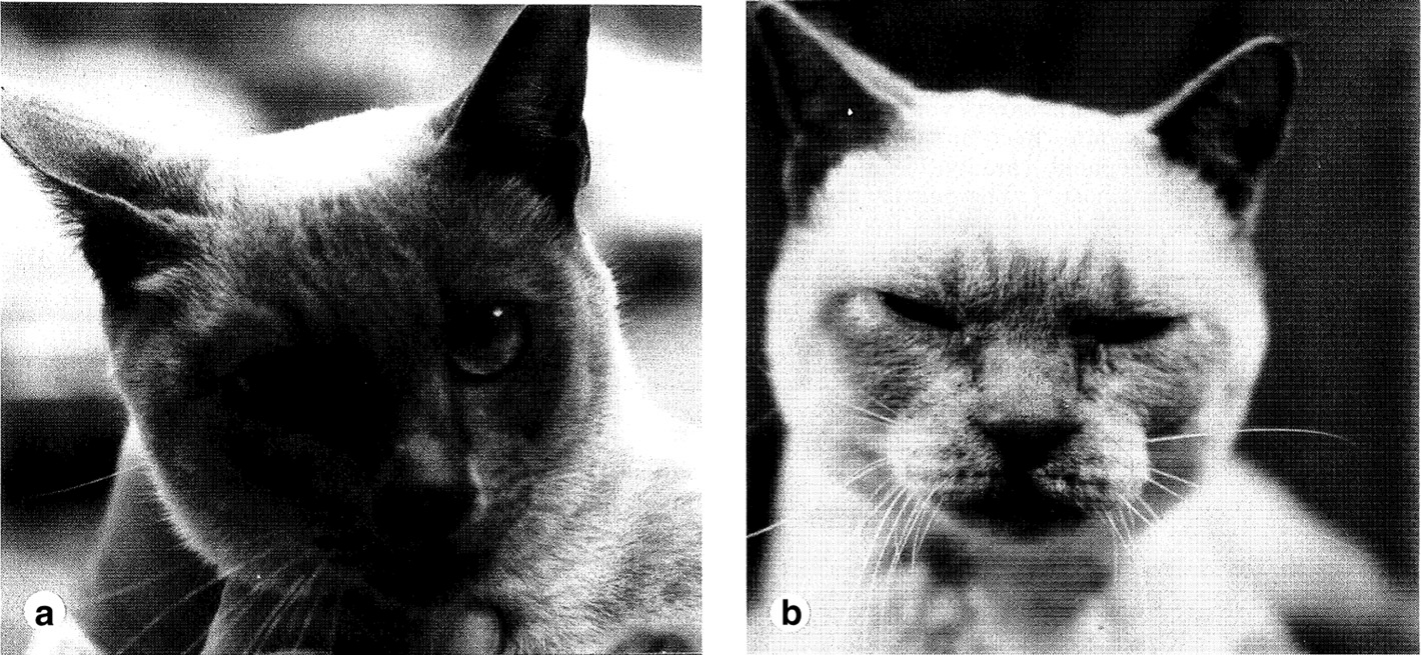
**a** Phenotypically normal Siamese dam of the affected cat in **b. b** Siamese affected cat with *ARSB* variant causing severe MPS VI disease. Notice the broad maxillary area, slightly flattened face and half-closed eyes. (Reproduced with permission from Cowell et al.*,* 1976.) [32]

**Fig. 2:**
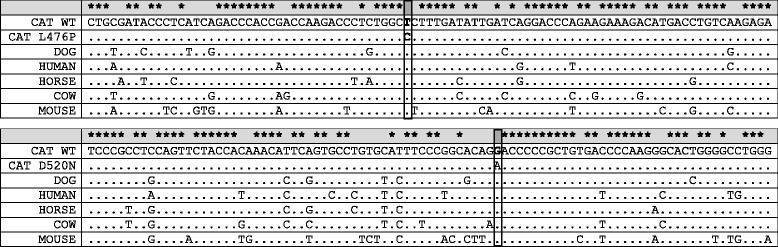
Alignment of *ARSB* DNA sequence fragments across mammals. Presented are the wildtype sequences for the domestic cat and other species and the cat sequences with variants at the nucleotide indicated with a box. The mutation sites are the L476P (C**T**C to C**C**C) and the D520N mutation (**G**AC to **A**AC). Note each mutation is on the wildtype sequence background and not compound heterozygotes. Both sites are conserved across mammals. Alignment produced by ClustalX software. The asterisks indicate conservation of the wildtype nucleotide across all species

Cowell et al., (1976) [32] reported the first case of an inherited disorder of mucopolysaccharide metabolism in the cat. Suzie, a 21-month-old blue point Siamese cat born of a mother-son mating, presented as “decidedly small” and “reluctant to walk”. Suzie’s head was “smaller than normal with an overall shortened and broadened appearance”. The maxilla was remarkably broadened and decreased in length’ (Fig. 1). The cat had bilateral pain at flection and extension of the joins with radiographic skeletal striking deformities, multifocal neurologic deficits, corneal clouding, retinal atrophy and significantly high concentration of mucopolysaccharide in the urine. A Siamese – Siamese x domestic shorthair cross experimental colony was established and more complete descriptions of pathologic and enzymologic findings were recorded (Fig. 2) [17, 33]. MPS VI has since been diagnosed in Siamese, [34–36] and domestic longhair [37] cats from different locations and all were reported as similarly affected (OMIA 000666-9685) [38]. In cats, autosomal recessive mutation consists of an amino acid substitution of a wild type leucine to a proline (L476P) in the arylsulfatase B enzyme, due to a thymine to cytosine transition (c. 1427 T > C) in *ARSB* [10].

A missense mutation (c.1558G > A), inherited independently from L476P and causing an amino acid substitution of the wild type aspartic acid to an asparagine at codon 520 (D520N) was identified in the same colony (Fig. 2) [11, 12]. The phenotype of the D520N variant in combination with the L476P variant was dubbed MPS VI “mild”. Skin fibroblasts from adult D520N/L476P compound heterozygous cats expressed approximately 3.1 % of normal levels of ARSB activity and D520N homozygous cats contained approximately 4.6 %. L476P homozygous fibroblasts contained very low levels of ARSB activity at 0.46 %. All 18 colony cats that were D520N homozygotes were evaluated radiographically up to 5 years of age. This small increase in residual *ARSB* activity is sufficient to prevent the occurrence of degenerative joint disease in D520N homozygous cats. However, L476P/D520N compound heterozygous cats can develop severe joint disease as they age [11]. One cat exhibited an atypical joint disease in the shoulder. Therefore, in the clinical situation, these animals would most likely be indistinguishable from normal Siamese cats, although more clinically observable joint lesions could potentially develop in older animals since the cats were followed only for 5 years [39].

A population screening for both L476P and D520N was conducted on Siamese cats worldwide [39]. The prevalence was investigated in 101 clinically normal cats from North America, South America, Europe and Australia. In this general Siamese population, the D520N allelic frequency was 11.4 % (Table 1), but L476P was absent, suggesting either low frequency or higher prevalence within breeding lines excluded by the sampling. The authors noticed that “the high prevalence in the general population of the D520N, which might correspond to an expected D520N/D520N genotype frequency of 1.2 % in the sample, is “consistent with an extensive presence of carriers, to the extent two D520N/D520N homozygous were detected”, suggesting Hardy-Weinberg equilibrium.

**Table 1:** Prevalence of feline *ARSB* variants in Siamese cats from the general Siamese population

Origins	No. Alleles tested	L476P	D520N	(Percent)
Bristol, England	10	0/10	0/10	
Dublin, Ireland	24	0/24	3/24^a^	(12.5 %)
Argentina	4	0/4	2/4^b^	(50 %)
The Netherlands	8	0/8	1/8	(12.5 %)
Melbourne, Australia	132	0/132	14/132^c^	(10.6 %)
Pennsylvania	24	0/24	3/24^c^	(12.5 %)
Total	202	0	23	(11.4)

(reproduced with permission from Crawley et al, 2003) [39]

^a^Two animals possibly related

^b^Possibly littermates

^c^One cat was a D520N homozygote

### Commercial MPS VI screening

Commercial screening has been offered for MPS VI “mild”, the D520N (c.1558G > A) variant. All data were generated as fee for service and not based on experimentation. Over 2200 cats have been genotyped and the variant was identified in 13 different breeds (Table 2), including, Abyssinian, Birman, British Shorthair, German Longhair, Oriental Shorthair, Ragdoll, Russian Blue, Russian White, Selkirk Rex, Siamese, and Tonkinese. Most of these breeds have South East Asian origins and the low occurrence in Western European breeds, such as British Shorthair, Ragdoll and Selkirk Rex is likely due to cross breeding. Interestingly, cats with South East Asian origins, such as Korat and Singapura, and cats bred from Abyssinians, such as Ocicats, were not found to have the variant in the populations tested.

**Table 2:** Allele frequency of MPS VI variants in cat breeds

		Frequency	
BREED	N	L476P	D520N
		Severe	“Mild”
Abyssinian	114	0.00	0.179
Birman	150	0.00	0.313
Birman^a^	231	0.00	0.359
Birman (Italy)^b^	51	0.00	0.284
British Shorthair	244	0.00	0.006
Burmese	2	0.00	0.250
German Longhair^a^	3	0.00	0.333
Oriental Shorthair	5	0.00	0.200
Ragdoll	530	0.00	0.066
Ragdoll^a^	95	0.00	0.131
Russian Blue	44	0.00	0.239
Russian White	4	0.00	0.125
Selkirk Rex	16	0.00	0.063
Siamese	24	0.00	0.104
Somali	65	0.00	0.146
Tonkinese	1	0.00	0.500
39 additional breeds	678	0.00	0.000

^a^Indicates requested test

^b^Cats tested at Vetogene in Milan, Italy. All other cats were tested at the Veterinary Genetics Laboratory in Davis, California, USA

Both the *ARSB* cat variants are variants on the Illumina 63 K iSelect Infinium DNA array for the cat. In a survey of approximately 2602 archival cat samples from various disease and phenotypic projects that were unrelated to MPS studies, only four domestic cats were identified as carriers for the severe variant, including a domestic shorthair, a Siamese, an Ocicat, and a Chartreux. This low rate could be within the genotyping error rate of the array. However, 435 were carriers and 103 were homozygous (3.5 %) for the D520N “mild” variant. A majority of the homozygous and heterozygous cats were Birmans, as well as many African and European wildcats – suggesting the variant segregates in the wildcat population and may be a common ancestral allele.

## Conclusion

Genetics is an extremely powerful tool for animal health care and management because mating of cats can be controlled. Using genetic testing, cats can be bred in a manner to prevent illnesses, eliminating problems before they exist and even eradicating a disease from the population. However, genetics must be used wisely, not causing unwarranted strife and concern or causing elimination of individuals from a breeding pool that could make valuable contributions. Thus, researchers have the responsibility to vigorously define DNA variants that cause health problems in cats.

In humans, customized newborn screening programs that are tailored to the ethnicity of the population are active worldwide, screening for dozens of disease causing variants [40]. These DNA variants cause health problems where the negative health outcomes can be thwarted if early intervention can be applied. Concurrent with the screening programs, the fields of medical genetics and genetic counselling developed. Over 70 different genetic variants have been identified in cats that confer coat colors, fur types, morphological attributes, blood type and diseases [2]. However, formal medical genetic counselling programs do not exist for companion animals, thus the researchers and DNA testing laboratories must fulfill this role for the owners of cats with genetic diseases.

An uncontrolled selection against D520N is an example of how genetic testing can be used unwisely. The reduction of the already quite low genetic variability in some pedigreed breeds, such as Birmans, [41–43] which also have a high frequency of the D520N variant, can reduce genetic diversity and perhaps facilitate the proliferation of more detrimental mutations. Thinking this mutation must do “something”, breeders tend to find any health condition, especially common problems due to overcrowding and stress, and make an unscientific and highly biased association with disease, including concerns that are not associated with *ARSB* variants.

## Summary

The relevance of a genetic trait in terms of pain and impediment to function should be considered when publishing a DNA variant that may ultimately be requested as a genetic test both by veterinary practitioners and breeders. Severe genetic dysfunctions need to be tested, monitored and eventually eradicated. Conversely, elimination of genetic polymorphisms weakly or not associated to undefined or mild phenotypic forms endanger the genetic pool of at risk breeds.

Only the MPS VI L476P (c.1427 T > C) variant in *ARSB* causes severe disease in cats. Because the variant was found in the DNA array survey of unbiased cat breed sampling, perhaps this disease-associated variant should be monitored in the cat breed populations, particularly breeds with Siamese derivations and outcrosses. However, the *ARSB* D520N (c.1558G > A) has no evidence of association with disease and the authors recommend breeders discontinue testing for this variant, unless in combination with the L476 variant. Testing for D520N should be considered in heterozygous L476P cats, however, the L476P variant itself is extremely rare in the general cat population and should not be tested unless a MPS disease is suspected. Selection against the D520N variant is not warranted in cat populations. Researchers and commercial genetic testing laboratories need to carefully examine the published literature and perhaps openly discuss issues with genetic testing at forums such as the International Society of Animal Genetics (ISAG; http://www.isag.us/) and with the appropriate researchers with vested interests in the given disease or trait. Veterinarians and breeders are encouraged to be proactive and support research with samples, clinical records and funds to decipher the causes of health concerns in their cats.

## Abbreviations

*ARSB*, *N-acetylgalactosamine-4-sulfatase*; GAG, glycosaminoglycan; HCM, hypertrophic cardiomyopathy; MPS VI, mucopolysaccharidosis type VI; *MYBPC3*, *myosin-binding protein C3;* OMIM, Online Mendelian Inheritance in Man
